# Biochemical and Molecular Characterization of a Thermostable Alkaline Metallo-Keratinase from *Bacillus* sp. Nnolim-K1

**DOI:** 10.3390/microorganisms8091304

**Published:** 2020-08-27

**Authors:** Nonso E. Nnolim, Lindelwa Mpaka, Anthony I. Okoh, Uchechukwu U. Nwodo

**Affiliations:** 1 SAMRC Microbial Water Quality Monitoring Centre, University of Fort Hare, Alice 5700, South Africa; lmpaka@ufh.ac.za (L.M.); AOkoh@ufh.ac.za (A.I.O.); UNwodo@ufh.ac.za (U.U.N.); 2 Applied and Environmental Microbiology Research Group (AEMREG), Department of Biochemistry and Microbiology, University of Fort Hare, Private Bag X1314, Alice 5700, South Africa

**Keywords:** Keratinase, chicken feather, biodegradation, thiol group, valorization, bioeconomy, *Bacillus* sp.

## Abstract

Keratinases are considerably gaining momentum in green technology because of their endowed robustness and multifaceted application potentials, such as keratinous agro-wastes valorization. Therefore, the production of novel keratinases from relatively nonpathogenic bacteria grown in agro-wastes formulated medium is cost-effective, and also imperative for the sustainability of thriving bioeconomy. In this study, we optimized keratinase production by *Bacillus* sp. Nnolim-K1 grown in chicken feather formulated medium. The produced keratinase (KerBNK1) was biochemically characterized and also, the keratinase-encoding gene (*kerBNK1*) was amplified and sequenced. The optimal physicochemical conditions for extracellular keratinase production determined were 0.8% (*w/v*) xylose, 1.0% (*w/v*) feather, and 3.0% (*v/v*) inoculum size, pH 5.0, temperature (25 °C) and agitation speed (150 rpm). The maximum keratinase activity of 1943.43 ± 0.0 U/mL was achieved after 120 h of fermentation. KerBNK1 was optimally active at pH and temperature of 8.0 and 60 °C, respectively; with remarkable pH and thermal stability. KerBNK1 activity was inhibited by ethylenediamine tetra-acetic acid and 1,10-phenanthroline, suggesting a metallo-keratinase. The amplified *kerBNK1* showed a band size of 1104 bp and the nucleotide sequence was submitted to the GenBank with accession number MT268133. *Bacillus* sp. Nnolim-K1 and the keratinase displayed potentials that demand industrial and biotechnological exploitations.

## 1. Introduction

Keratins are structural proteins that offer mechanical support at the sites of occurrence [1]. Their abundance in agro-wastes particularly avian feathers generated from poultry processing farms prevents the efficient management of these byproducts [2], and consequently, they constitute variable degrees of environmental nuisance through their disposal means. 

The wealth of proteins locked-up in feathers and a huge stream of these wastes emanating from poultry processing farms had previously prompted traditional means of converting these materials into feedstuff, through endergonic and pressure treatment, accompanied by grinding into fine powder [3]. This product provides limited nutritional requirements when utilized as a feed supplement due to poor biodegradability and loss of essential heat-labile nutrients. More so, strong acid or alkali hydrolysis has been employed for the conversion of feathers into products with high beneficiation [4]. This approach not only affects the quality of the protein hydrolysates produced, but also elicits environmental issues.

Alternatively, bioconversion of feathers has presented greater fortunes as it yields important products with many application potentials [4,5]. Prior to the isolation and characterization of a throng of keratinolytic bacteria including the most extensively studied *Bacillus licheniformis* PWD-1 [6], keratinolytic activity was predominantly observed among fungi as the mechanism of their host’s infiltration [7]. However, the pathogenic nature of most keratinolytic fungi has been a bottleneck to their prospective commercialization; consequently, keratinolytic bacteria exploration has been on the upward trajectory. 

Many keratinolytic bacteria have been identified to date with variable feather-degrading capacity. Some keratinolytic bacteria and their respective feather biodegradation timeline include *Chryseobacterium* sp. RBT after 48 h [8], *Bacillus subtilis* after 72 h [9], *Streptomyces* sp. S7 after 96 h [10], and *B. licheniformis* PWD-1 after 10 days [6]. The biodegradation of feathers by keratinolytic microbes is mediated through the expression and extracellular production of keratinase. The degree of feather degradation is contingent on the enzyme yield and catalytic efficiency of the produced keratinolytic enzyme [2]; however, not all the peptidases could efficiently liberate soluble proteins from keratin even after structural alteration by sulfitolytic system [11].

Keratinases are promising candidates in many sectors of bioeconomy due to their robustness and catalytic tendency [12]. Profuse keratinase research activity has been implemented for about three decades, however, only a few of these enzymes, predominantly from strains of *B. licheniformis* have been commercialized [13,14]. Therefore, sourcing for novel keratinases from efficient bacterial producers and establishing optimal physicochemical conditions for their scale-up production purposes are imperative. 

Several optimization approaches have been adopted, including classical and statistical methods. Optimization of keratinase production by bacteria is crucial due to strain variations. The classical method considers one variable at a time (OVAT). This method is effective in the selection of significant variables; however, it is tedious and also fails to give the interaction patterns of the considered parameters [15]. Response surface methodology (RSM) gives the interactions of significant variables and limits the number of experimental runs, and hence, is a time-saving process. However, statistical optimization depends on the classical method for its adequacy as an arbitrary selection of the level of study may meet a dead end due to strain peculiarity [16]. Therefore, the combination of these two approaches may effectively influence microbial productivity. 

Keratinases are swiftly gaining industrial and biotechnological attention, and may find significant application potential in feed production, leather processing, the detergent formulation in the view of amelioration of the environmental hazard, and waste burdens [17,18]. It is worthy to note that the successful application of an enzyme is contingent on the optimal biochemical characteristics. Therefore, this study was undertaken to optimize the physicochemical conditions for extracellular keratinase production by *Bacillus* sp. Nnolim-K1 grown in chicken feather formulated medium. Additionally, the keratinase biochemical properties such as pH and temperature optima and stability, the effect of chemical agents and metal ions, impact of commercial laundry detergent, and keratinase gene fragment of *Bacillus* sp. Nnolim-K1 was characterized accordingly. 

## 2. Materials and Methods

### 2.1. Preparation of Keratin Substrate

Preparation of powdered chicken feather (PCF) substrate utilized in this study followed a similar approach as we reported previously [19].

### 2.2. Bacterial Isolate Selection 

Chicken-feather-degrading *Bacillus* sp. Nnolim-K1 utilized for this study was previously isolated from a soil sample collected from a municipal dumpsite [20], and maintained on PCF agar slant in the culture repository of Applied and Environmental Microbiology Research Group (AEMREG). 

### 2.3. Bacterial Resuscitation and Fresh Inoculum Preparation

Basal salt medium (BSM) containing K_2_HPO_4_, 0.3 g; KH_2_PO_4_, 0.4 g; MgCl_2_, 0.2 g; CaCl_2_, 0.22 g and PCF, 10 g/L of distilled water [19], was inoculated with pure bacterial isolate from PCF agar slant and incubated for 48 h at 30 °C under agitation (130 rpm). Thereafter, a loopful of the culture broth was streaked on a PCF agar plate. PCF agar plate contained the BSM with the addition of 15 g/L of bacteriological agar. The plate was incubated for 24 h at 30 °C. Subsequently, some bacterial colonies were harvested into a microtube containing sterile saline (8.5 g/L NaCl), and washed twice. The resulting pellet was resuspended in sterile saline and the optical density was adjusted to 0.1 at 600 nm and this served as a fresh inoculum for the subsequent fermentation process. 

### 2.4. Keratinase Production

Keratinase production was carried out in 500 mL Erlenmeyer flasks containing about 97 mL of BSM that comprised g/L: K_2_HPO_4,_ 0.3; KH_2_PO_4_, 0.4; MgCl_2_, 0.2; CaCl_2_, 0.22, PCF, 10; xylose, 8. The flasks were autoclaved (Already Enterprise Inc., Beitou District, Taipei City, Taiwan) and the pH of the media was aseptically adjusted to 5 with the JENWAY pH meter (Bibby Scientific Ltd., Stone, Staffordshire, UK). Thereafter, the flasks were inoculated with 3% (*v/v*) of the fresh bacterial suspension and incubated in a rotary shaker (Labotec, Midrand, Gauteng, South Africa) for 120 h at 25 °C and 150 rpm. After the fermentation period, cell biomass and undegraded feathers were harvested by centrifugation (Beckman Coulter Inc., Carlsbad, CA, USA) at 15,000 rpm for 15 min and 4 °C. Supernatant that was recovered served as crude keratinase and was utilized for the subsequent analysis.

#### 2.4.1. Keratinase Activity Assay and Quantitation of Total Protein

Assay of keratinase activity using the crude extract was in accordance with the method of Jaouadi et al. [21], with slight modification as previously described [19]. Briefly, the reaction mixture contained 0.5 mL of 10 g/L of keratin azure (Sigma-Aldrich, St. Louis, MO, USA) in 0.1 M Tris-HCl buffer, pH 8.0, and 0.5 mL of crude enzyme solution. The mixture was incubated at 60 °C for 1 h, with shaking at 220 rpm; thereafter, the reaction was stopped by placing the assay mixture in ice-cooled water for 10 min. The unutilized substrates were removed by centrifugation at 15,000 rpm for 10 min, and subsequently filtered (Millipore cellulose filters; 0.45 μm, Merck chemicals (Pty) Ltd., Modderfontein, Gauteng, South Africa). The azo dye released in the filtrate was determined at 595 nm, using a SYNERGYMx 96-well microplate reader (BioTek Instrument Inc., Winooski, VT, USA). The control was treated at the same condition which contained the enzyme solution and buffer without the substrate. One unit of keratinase activity (U) was defined as the amount of enzyme causing an increase of 0.1 in absorbance per hour under the standard assay condition. 

Total protein concentration in the test sample was quantitated by extrapolating from the standard protein curve constructed using varying concentrations of bovine serum albumin in accordance with the Bradford method [22]. 

#### 2.4.2. Determination of Thiol Concentration

The thiol group concentration in the cell-free broth was quantified in accordance with the Ellman method [23].

### 2.5. Construction of Optimal Process Variable for Enhanced Keratinase Production

#### 2.5.1. One Variable at a Time (OVAT) Method

##### Effect of Initial Medium pH, Incubation Temperature and Agitation Speed

The effect of initial medium pH on keratinase production by *Bacillus* sp. Nnolim-K1 was investigated by varying the initial pH value of the BSM from pH 4.0 to pH 11.0, at 1 unit interval. Similarly, the effect of temperature was studied by varying the incubation temperature from 25 °C to 40 °C, at a 5 °C interval. The effect of agitation speed on keratinase production by *Bacillus* sp. Nnolim-K1 was evaluated by performing the submerged state fermentation from 0 to 200 rpm, at an interval of 50 rpm. 

##### Effect of Extra Carbon and Nitrogen Supplementation

The effect of carbon source supplementation on keratinase production by *Bacillus* sp. Nnolim-K1 was studied by supplementing the fermentation medium with the following saccharides (xylose, mannitol, glucose, fructose, sucrose, maltose, soluble starch, galactose, lactose, and sorbitol; Merck Chemicals (Pty) Ltd., Modderfontein, Gauteng, South Africa) at a final concentration of 0.1% (*w/v*). Similarly, 0.02% (*w/v*) of different nitrogen sources such as yeast extract, beef extra, malt extract, casein, gelatin, tryptone, urea, peptone, (NH4)_2_SO_4_, NH_4_NO_3_, KNO_3_, NaNO_3_, NH_4_Cl, (NH_4_)H_2_PO_4_, and (NH_4_)_2_HPO_4_ (Merck chemicals (Pty) Ltd., Modderfontein, Gauteng, South Africa), was individually added to the fermentation medium. The optimal concentration of the best carbon source was determined by varying the concentration from 0.1% to 0.8%.

##### Effect of Chicken Feather Concentration 

Chicken feather was added to the fermentation medium between 0.1% and 1.75% (*w/v*) to determine the optimal concentration for the maximum extracellular keratinase production by *Bacillus* sp. Nnolim-K1. 

#### 2.5.2. Statistical Optimization

##### Experimental Design and Response Surface Methodology (RSM)

The effect of four significant independent variables (xylose concentration, initial pH, feather concentration, and inoculum size) was further optimized using response surface methodology. In total, 30 experimental runs were generated using central composite design (CCD) of RSM in Design-Expert Software Trial Version 11.1.0.1 (Stat Ease Inc., Minneapolis, MN, USA), and each variable in the design was studied at three different levels (−1, 0, 1; Table 1). The ranges of the variables and complete experimental plan in relation to their values and coded forms are listed in Table 2. The experiments were carried out in triplicate and upon completion; the data obtained were fitted to the second-order polynomial equation by multiple regression analysis to predict the response (*Y*) of the variables. This resulted in an empirical model that relates the measured response to the independent variables of the experiments as shown in Equation (1).

(1) $$Y=\beta_{0}+\overset{n}{\underset{i=1}{\sum }}\beta i\chi i+\overset{n}{\underset{i=1}{\sum }}\beta ii\chi i^{2}+\overset{n=1}{\underset{i=1}{\sum }}\neq \overset{n}{\underset{j=i+1}{\sum }}\beta ij\chi i\chi j+\varepsilon$$

where *Y* is the predicted response; *β₀* is the intercept; βi, *β*ii, and βij are the coefficient values for linear, quadratic and interaction effects, respectively; χi and χj are the coded forms of the input variables; ε and *n* are the standard error and number of independent variables, respectively.

#### 2.5.3. Time Course Profile of Keratinase Activity

Time course profile of keratinase activity by *Bacillus* sp. Nnolim-K1 was studied using an optimized medium that comprised (*w/v*): 0.03% K_2_HPO_4_, 0.04% KH_2_PO_4_, 0.02% MgCl_2_, 0.022% CaCl_2_, 0.8% xylose, 1% PCF. The fermentation was carried out using 250 mL Erlenmeyer flasks containing 97 mL of BSM, and the initial pH was aseptically adjusted to 5.0 after sterilization. Subsequently, the flasks were inoculated with 3% (*v/v*) bacterial suspension, and incubated for 192 h at 25 °C under agitation (150 rpm). Aliquots were aseptically and periodically (24 h interval) withdrawn to determine the keratinase activity vis-à-vis keratinase production, medium pH change, cell growth, thiol, and total protein concentrations. 

### 2.6. Biochemical Characterization of Keratinase (KerBNK1) 

#### 2.6.1. Effect of pH and Temperature on KerBNK1 Activity and Stability

The effect of pH on keratinase activity was evaluated using the following buffer solutions (0.1 M): sodium citrate buffer (pH 5.0), potassium phosphate buffer (pH 6.0–7.0), Tris-HCl buffer (pH 8.0–9.0), and sodium bicarbonate-NaOH (pH 10.0–11.0). The pH stability of the enzyme was evaluated by preincubating the enzyme with different buffer solutions (pH 6.0–9.0) for 5 h at 30 °C. Aliquots were periodically withdrawn (1 h interval) and the residual activity was determined using the standard assay protocol. The enzyme–buffer solution without preincubation served as the control experiment. 

The effect of temperature on keratinase activity was investigated by varying the assay temperatures from 30 °C to 80 °C, at an interval of 5 °C. Thermal stability of the keratinase was studied by subjecting the enzyme solution to thermal pretreatment from 55 °C to 75 °C for 150 min using TECHNE Dri-Block DB-3D (Bibby Scientific Ltd., Staffordshire, UK). Again, aliquots were periodically withdrawn (30 min interval), and used to determine the residual enzyme activity at standard assay protocol. The enzyme solution without preheating served as the control experiment. 

#### 2.6.2. Effect of Chemical Agents on KerBNK1 Stability

The impact of some groups of chemical agents such as protease inhibitors (5 mM): phenylmethylsulfonyl fluoride (PMSF), ethylenediamine tetra-acetic acid (EDTA), 1,10-phenanthroline (Sigma-Aldrich, St. Louis, MO, USA); reducing agents (5 mM): sodium thioglycolate (C_2_H_3_NaO_2_S), dithiothreitol (DTT), sodium sulfite (Na_2_SO_3_), 2-mercaptoethanol (2-ME); hydrogen peroxide (H_2_O_2_; 1%, *v/v*); organic solvent (1%; *v/v*): dimethyl sulfoxide (DMSO), acetonitrile, propan-2-ol; nonionic surfactants (1%; *v/v*): triton X-100, tween-80; and anionic surfactant: sodium dodecyl sulfate (5 mM) on enzyme stability was investigated by preincubating the enzyme solution with the various agents for 1 h at 30 °C. Thereafter, the residual keratinase activity was determined under the standard assay protocol. The enzyme solution preincubated with distilled water following similar conditions served as the control and was taken as 100%. 

#### 2.6.3. Effect of Metal Ions on KerBNK1 Stability

The enzyme solution was preincubated with various metal salts including LiCl, NaCl, AgCl, KCl, CaCl_2_, MgCl_2_, MnCl_2_, ZnCl_2_, CuCl_2_, FeCl_2_, HgCl_2_, BaCl_2_, CoCl_2_, AlCl_3_, and FeCl_3_ (Merck chemicals (Pty) Ltd., Modderfontein, Gauteng, South Africa) at a final concentration of 5 mM for 1 h. Subsequently, residual enzyme activity was determined using standard assay protocols. The enzyme solution preincubated with distilled water served as the control and was taken as 100%.

### 2.7. Molecular Characterization of KerBNK1

#### 2.7.1. DNA Extraction 

*Bacillus* sp. Nnolim-K1 genomic DNA was extracted using the boiling method [24]. Briefly, some bacterial colonies of 18 h culture were suspended in 200 µL sterile nuclease-free water and homogenized by vortexing. The homogenous bacterial suspension was boiled at 100 °C for 10 min using TECHNE Dri-Block DB-3D (Bibby Scientific Ltd., Staffordshire, UK). Subsequently, the solution was centrifuged for 5 min at 13,500 rpm (HERMLE Labortechnik GmbH, Siemensstr, Wehingen, Germany). The supernatant was transferred into another sterile vial and utilized as a DNA template for the polymerase chain reaction (PCR) assay.

#### 2.7.2. Keratinase Gene Amplification 

The gene fragment of *Bacillus* sp. Nnolim-K1 encoding keratinase was amplified by conventional PCR using a set of newly designed oligonucleotides; *kerBNK1*F: TCATCTACTGATTACGTTCC and *kerBNK1*R: TTAAGAAGCTTTATTTTCTTG as forward and reverse primers, respectively. The primers were designed by using keratinase gene sequences from the *Bacillus cereus* group available in the National Center for Biotechnology Information (NCBI) and synthesized by Inqaba Biotechnical Industries (Pty) Ltd., Muckleneuk, Pretoria, Gauteng Province, South Africa. The PCR was carried out using 25 µL reaction mixtures which consisted of 12.5 µL of OneTaq^®^ Quick-Load^®^ 2X master mix (New England Biolabs Inc., Pretoria, South Africa), 5.5 µL of nuclease-free water, 1 µL each of both forward and reverse primers and 5 µL of DNA template. The target gene amplification was done using T100™ Thermal Cycler (Bio-Rad Laboratories Inc., Singapore, Singapore), under the following cycling conditions: initial denaturation at 95 °C for 5 min, 35 cycles of denaturation at 95 °C for 30 s, annealing at 50 °C for 1 min, extension at 72 °C for 1 min, and a final extension at 72 °C for 5 min. The amplicon was electrophoresed on ethidium-bromide-stained 1.2% agarose gel (Merck chemicals (Pty) Ltd., Muckleneuk, Pretoria, Gauteng, South Africa), and subsequently, visualized under an ultraviolet transilluminator (Uvitec, Cambridge, UK). 

#### 2.7.3. Sanger Sequencing and Phylogenetic Analyses

The PCR products were analyzed using the dideoxynucleotide chain termination method [25]. Prior to sequencing reaction, the amplified products were subjected to a post PCR clean-up using the Nucleofast 96-well post PCR clean-up plate (Macherey Nagel GmbH & Co., Valencienner, Düren, Germany) on a Tecan EVO150 robotic workstation (Freedom EVO^®^, Männedorf, Zürich, Switzerland), following the manufacturer’s protocol. The samples were denatured for 2 min at 95 °C and then cooled to 4 °C. Subsequently, the purified PCR products were sequenced using the BigDye Terminator V3.1 sequencing kit (Applied Biosystems, Foster City, CA, USA) on an ABI3730XL DNA Analyzer (Foster City, CA, USA) using a 50 cm capillary array and POP7 (Applied Biosystems, Foster City, CA, USA) following the protocols supplied by the manufacturer. The PCR products were sequenced in both directions to obtain a reliable sequence.

The sequencing results were analyzed using Geneious Prime V2020.1.1 (Biomatters Ltd., Auckland CBD, Auckland, New Zealand) and the nucleotide sequence was submitted to the GenBank with the accession number MT268133. A BLAST search was conducted to retrieve similar sequences from the NCBI (https://blast.ncbi.nlm.nih.gov/Blast.cgi). Phylogenetic analysis of the keratinase gene sequence was conducted in MEGA7 [26]. Furthermore, multiple keratinase sequence alignment was performed using the ClustalW program in BioEdit [27], as the physicochemical properties including, molecular weight (MW), theoretical isoelectric point (pI), instability index, and aliphatic index were computed using ExPASy-ProtParam tool (https://web.expasy.org/protparam/) [28].

### 2.8. Evaluation of Laundry Detergent Impact on KerBNK1 Stability 

The impact of some selected commercially available laundry detergents on the stability of keratinase was evaluated using the method previously described by Paul et al. [17]. Briefly, the solid detergents which include Sunlight, Omo, Surf (Unilever, Durban, KwaZulu-Natal, South Africa), Ariel (Procter and Gamble, Sandton, Johannesburg, Gauteng, South Africa) and Maq (Bliss Brands (Pty) Ltd., Johannesburg, Gauteng, South Africa) were dissolved in tap water to a final concentration of 7 mg/mL and heated for 30 min at 100 °C to inactivate the endogenous enzymes. The enzyme solution was mixed with the pretreated detergent in a ratio of 4:1. The mixture was incubated for 60 min at 40 °C and the residual enzyme activity was measured according to the standard assay protocol. The enzyme solution preincubated with tap water only served as the control and was taken as 100%.

### 2.9. Biodegradation of White and Melanized Feathers—Structural Evaluation 

The ability of *Bacillus* sp. Nnolim-K1 to degrade white and melanized feathers (1%, *w/v*) was investigated using optimized BSM. The medium was inoculated with 3% (*v/v*) bacterial suspension and incubated for 48 h at 25 °C and 150 rpm. Thereafter, the degree of feather degradation and structural destruction were assessed by following the method we described previously [19]. 

### 2.10. Statistical Analysis

All experiments and sample analyses were performed in triplicate. The data were subjected to analysis of variance and compared at a *p* < 0.05 significant level. Statistical Package for Social Science version 23 (Armonk, NY, USA) was used for the analysis. 

## 3. Results and Discussion

### 3.1. Optimization of Keratinase Production by Bacillus sp. Nnolim-K1

#### 3.1.1. Selection of Significant Physicochemical Parameters using OVAT

**Figure 1:**
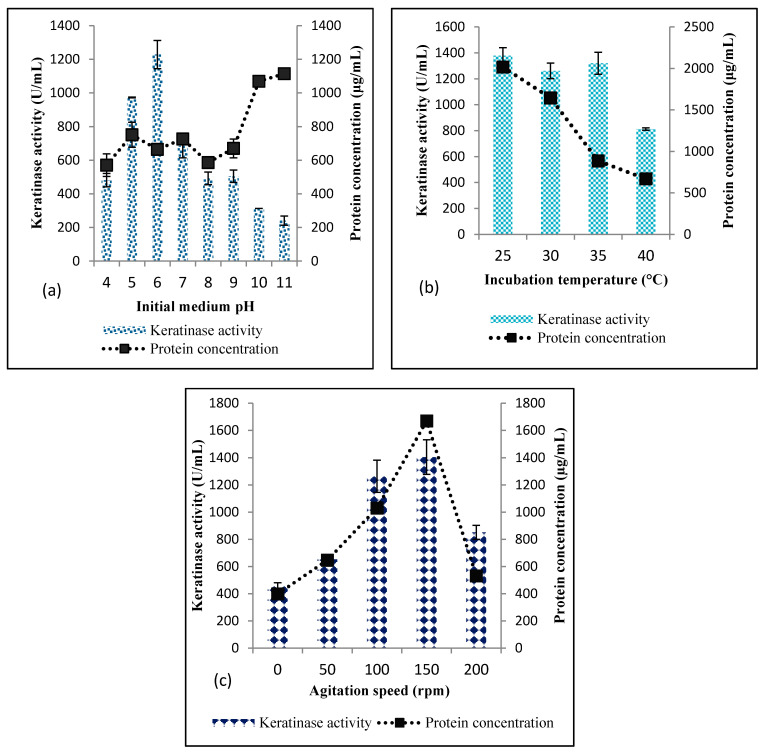
Effects of (**a**) initial medium pH (**b**) incubation temperature (**c**) agitation speed on keratinase production by *Bacillus* sp. Nnolim-K1. Each point represents the mean and standard deviation of triplicate experiments.

*Bacillus* sp. Nnolim-K1 was cultivated in BSM with different initial pH values that ranged from 4.0 to 11.0. The results showed that this isolate produced keratinase at all the pH tested, with an optimal keratinase activity of 1228.18 ± 83.56 U/mL at pH 6.0 (Figure 1a). The isolate showed significant keratinase activity at pH 4.0 (480.92 ± 39.85 U/mL), and further doubled at pH 5.0 (972.73 ± 2.57 U/mL). Beyond pH 6.0, the enzyme secretion by strain Nnolim-K1 declined significantly. The enzyme production was considerably reduced at pH 10.0 and pH 11.0 with a keratinase yield of 312.73 ± 0.0 U/mL and 240.91 ± 26.99 U/mL, respectively. This finding is similar to the reported pH optima for the production of keratinase by *Bacillus* spp. [19,29]. 

On the contrary, total protein concentration quantified from the cell-free supernatant increased significantly as pH drifted to strong alkaline conditions, with pH 10.0 and pH 11.0 recording the highest protein concentrations of 1069.65 ± 18.88 µg/mL and 1114.6 ± 0.0 µg/mL, respectively. The cause of increased protein concentration at strong alkaline is not clear. Nonetheless, it might be that the isolate produced other proteins that were not keratinase at this pH. Alternatively, alkaline conditions could promote the hydrolysis of feather keratin, hence, liberating quantifiable soluble proteins [4].

The incubation temperature of culture flasks was varied from 25 °C to 40 °C. The results revealed that *Bacillus* sp. Nnolim-K1 produced keratinase at mesophilic conditions, with an optimal temperature of 25–35 °C (Figure 1b). Beyond 35 °C, the enzyme activity of strain Nnolim-K1 decreased significantly, with an extracellular keratinase activity of 811.82 ± 8.99 U/mL. This finding is consistent with some documented reports [30,31]. However, due to strains variable peculiarities, some keratinolytic bacteria have been reported to present optimal keratinase activity between 45 °C and 70 °C [32,33]. 

Remarkably, total protein concentration decreased consistently from 2016.66 ± 3.34 µg/mL, 1643.99 ± 13.37 µg/mL, 885.24 ± 23.39 µg/mL, to 669.36 ± 5.57 µg/mL, with increasing incubation temperature from 25 °C, 30 °C, 35 °C to 40 °C, respectively. The reduction of total protein as the incubation temperature increased suggests that the production of protein metabolites by *Bacillus* sp. Nnolim-K1 is temperature-sensitive [19]. Therefore, keratinase production by *Bacillus* sp. Nnolim-K1 under mesophilic conditions indicates an energy-saving process which is highly sustainable from an economic perspective.

The influence of agitation condition on keratinase production by *Bacillus* sp. Nnolim-K1 was investigated from 0 to 200 rpm (Figure 1c). The results showed that keratinase production by strain Nnolim-K1 increased consistently with increasing agitation speed from 0 rpm (454.55 ± 25.71 U/mL) to 150 rpm (1404.54 ± 127.28 U/mL). However, keratinase activity at 100 rpm and 150 rpm was insignificant (*p* > 0.05). Beyond 150 rpm, keratinase production decreased significantly, with an enzyme activity of 840.91 ± 51.43 U/mL at 200 rpm. 

A similar trend was also observed for total protein concentration. Culture fermentation under agitation promotes aeration and homogeneous mixing of the medium components, which subsequently encourage growth and metabolism of the microorganism. However, agitation speed exceeding the optimal limit may impact negatively on the microbial cells through shear forces, hence decreasing their productivity. Similarly, agitation speeds ranging from 120 to 150 rpm optimally promoted keratinase production by some bacterial strains [19,34,35]. Contrarily, *Bacillus* sp. JB 99 demonstrated maximum keratinase production under constant shaking at 180 rpm [36]. Additionally, 200 rpm was well adapted for optimal activity by some keratinolytic *Bacillus* spp. [18,37]. 

**Figure 2:**
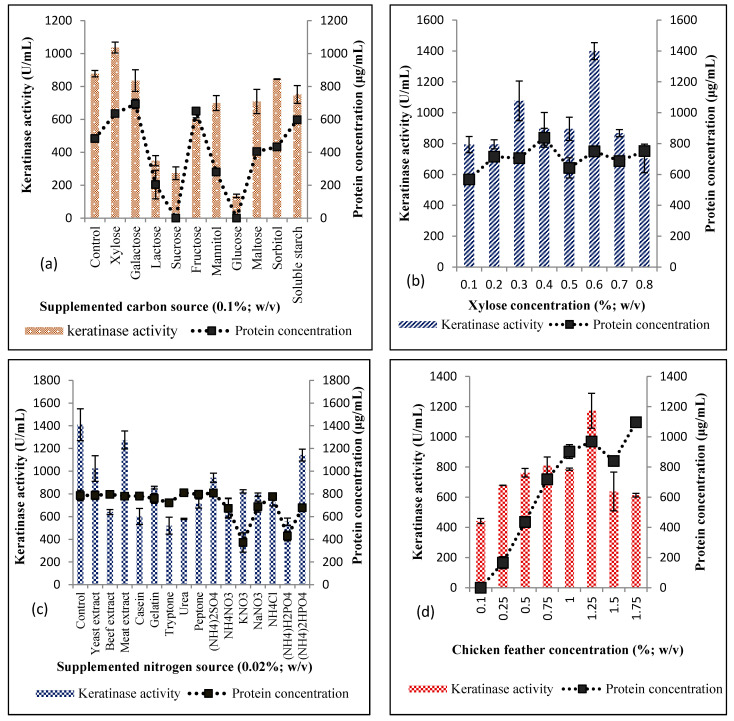
Effects of (**a**) saccharides supplementation, (**b**) xylose concentration, (**c**) nitrogen source supplementation, and (**d**) chicken feather concentration on keratinase production by *Bacillus* sp. Nnolim-K1. Each point represents the mean and standard deviation of triplicate experiments.

Supplementation of BSM with different carbon sources other than chicken feather showed that xylose significantly increased the enzyme production (1036.37 ± 33.43 U/mL) when compared with the control (877.28 ± 19.28 U/mL; Figure 2a). The presence of galactose and sorbitol did not show any significant alteration (*p* > 0.05) of enzyme activity when compared with the control. However, other carbon sources tested presented a variable repressive effect on the keratinase production by *Bacillus* sp. Nnolim-K1, and a more profound inhibition was exhibited by lactose, sucrose, and glucose, with the respective keratinase activity of 349.09 ± 30.86 U/mL, 272.73 ± 38.57 U/mL, and 134.55 ± 10.29 U/mL. Xylose concentration was further varied from 0.1% to 0.8% (*w/v*), and the results showed that 0.6% was optimum concentration for keratinase production by *Bacillus* sp. Nnolim-K1 as it presented extracellular enzyme activity of 1399.09 ± 55.28 U/mL (Figure 2b). The exact mechanism of which xylose promoted keratinase production is not clear. Nonetheless, we submit that upregulation of the keratinase-encoding gene by xylose operon activation may have played a significant role [19,38]. This finding is comparable to the keratinase activity by *Bacillus amyloliquefaciens* 6B, where supplementation of BSM with 0.5% (*w/v*) xylose promoted enzyme yield [39]. Repression of enzyme production has been a common attribute of saccharides among which glucose is a major culprit [30,40]. 

BSM was supplemented with different nitrogen (organic and inorganic) sources at a concentration of 0.02% (*w/v*). The results showed that keratinase production by *Bacillus* sp. Nnolim-K1 did not increase with the addition of nitrogen sources when compared to the control (Figure 2c). *Bacillus* sp. Nnolim-K1 showed optimal enzyme yield in the control with an extracellular keratinase activity of 1409.99 ± 140.14 U/mL, although there was no significant difference (*p* > 0.05) between enzyme yield in control experiment and that of a medium supplemented with malt extract which presented a keratinase activity of 1274.54 ± 79.91 U/mL. Total protein concentration determined in the various media was fairly constant across the board, except for KNO_3_ and (NH_4_)H_2_PO_4_ that had lower protein concentration of 372.32 ± µg/mL and 419.05 ± 42.34 µg/mL, respectively. Understandably, the low enzyme yield in the presence of nitrogen source and feathers indicated that the microorganism preferred easily utilizable nitrogen sources, hence the drop in the keratinase production. 

This finding is in accord with the report of Gurav and Jadhav [32], where the keratinase production by *Chryseobacterium* sp. RBT was inhibited in the presence of extra sources of nitrogen tested. Contrariwise, keratinase production by *Bacillus* spp. has been promoted by supplementation of BSM with 0.1% (*w/v*) NH_4_Cl [34] and 2 g/L yeast extract [41]. This could perhaps indicate that conventional nitrogen source utilization for optimum keratinase production by microorganisms is strain specific. The ability of *Bacillus* sp. Nnolim-K1 to preferentially utilize feather wastes for the optimum elaboration of keratinase portends its waste valorization and management tendencies. 

The BSM chicken feather concentration effect on keratinase production was evaluated from 0.1% to 1.75% (*w/v*). The obtained results showed that enzyme production increased with increasing chicken feather concentration, and peaked at 1.25% chicken feather with a keratinase activity of 1172.73 ± 115.71 U/mL (Figure 2d). Beyond 1.25%, keratinase production decreased significantly, with an enzyme yield of 638.18 ± 128.57 U/mL and 613.64 ± 11.57 U/mL at 1.5% and 1.75% of feather concentration, respectively. Additionally, total protein concentration increased consistently as feather concentration increased with a maximum concentration of 1097.19 ± 6.69 µg/mL at 1.75% of chicken feather. However, a slight drop in the protein concentration was observed at 1.5% feather concentration (840.33 ± 6.69 µg/mL) when compared to 1.25% feather concentration (969.55 ± 8.91 µg/mL). Extracellular keratinase production by microorganisms is generally inducible when keratinous substrates are available as a primary nutritional source. The level of keratinase expression by *Bacillus* sp. Nnolim-K1 may be commensurate to chicken feather concentration. Nonetheless, higher feather concentration instigated a reduction of keratinase production which might reasonably imply that more assimilable products of feather degradation amassed the medium. This would, as expected, lead to the downregulation of the keratinase-encoding gene [19]. The high viscosity of the medium at higher concentration feathers has been put forward as a possible reason for decreased keratinase production [39,42]. Since the optical density was not presented along with the results of other analyzed parameters during physicochemical conditions optimization, it will be pertinent to say that the observed fluctuations in total protein concentration and keratinase activity might also be attributed to variation in microbial cell growth at different factors considered.

#### 3.1.2. Optimization by Response Surface Methodology (RSM)

Four factors including xylose concentration, initial pH, feather concentration, and inoculum size were further optimized using RSM. CCD was used to evaluate the keratinase production by *Bacillus* sp. Nnolim-K1 in 30 experimental runs, and the experimental and predicted response values are presented in Table 2. The maximum keratinase production of 1589.09 U/mL was achieved at Run 5 that comprised 0.8% (*w/v*) xylose (A), pH 5.0 (B), 1% (*w/v*) feather (C), and 3% (*v/v*) inoculum (D) predicted by CCD to be 1460.64 U/mL. Subsequently, the experimental data were submitted to regression analysis and the second-order polynomial for keratinase activity obtained is as shown in Equation (2):

(2) $$\mathrm{Keratinase}\;\mathrm{activity}=1232.85+8.28\;A-81.82\;B-55.50\;C+23.64\;D-50.11\;\mathrm{CD}-\;62{.19\;A}^{2}+29{.63\;B}^{2}+40{.99\;C}^{2}$$

**Figure 3:**
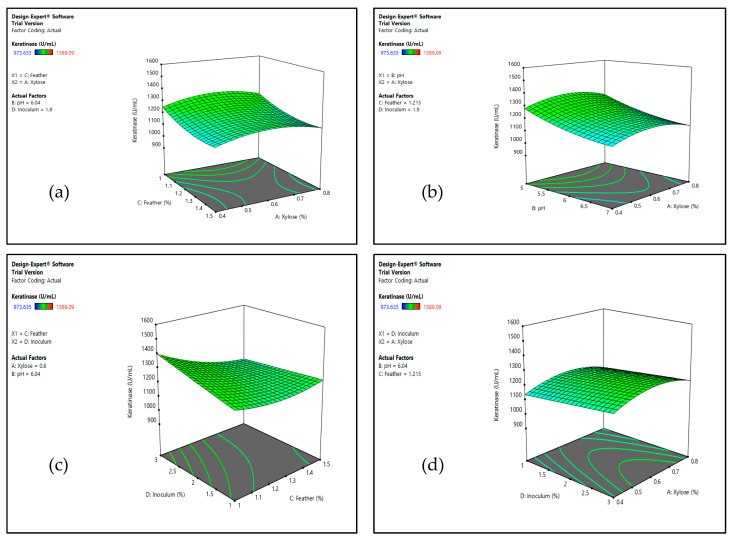
The 3D plot showing the interaction between (**a**) feather and xylose concentration, (**b**) pH and xylose concentration, (**c**) inoculum size and feather concentration, and (**d**) inoculum size and xylose concentration on keratinase activity by *Bacillus* sp. Nnolim-K1.

The model was significant for keratinase production (*p* < 0.05), as indicated by the nonsignificant lack of fit from the ANOVA (data not shown) which is generally desirable. Independent variables in a linear form such as B, C, and the quadratic term CD were significant contributors to the observed keratinase activity (Table 2). Furthermore, interaction patterns of the four variables were studied using response surface three dimensional (3D) curves (Figure 3). The 3D plots predicted that a decrease in feather concentration and initial pH value, with increasing inoculum size, presented a positive influence on the enzyme yield. However, the concentration of xylose had no significant contribution to the overall process. Keratinase is an inducible enzyme and it is amply elaborated by microorganisms in a medium containing keratinous material [19]. Nonetheless, an increase in the medium concentration of keratinous biomass beyond the optimal level has been reported to impact negatively on extracellular keratinase secretion by microorganisms [43,44]. Following the predicted significance of initial pH, feather concentration, and inoculum size from the interaction studies, three random experiments were performed, and the observed keratinase activity ranged from 1438.64 U/mL to 1625.31 U/mL. This, therefore, validated the aptness of the model for keratinase production by *Bacillus* sp. Nnolim-K1. This result is comparable to that reported for keratinase production by *B. licheniformis* ER-15 in a relatively modified medium tagged FM4 [16].

#### 3.1.3. Time Course Profile of Keratinolytic Activity by *Bacillus* sp. Nnolim-K1 

**Figure 4:**
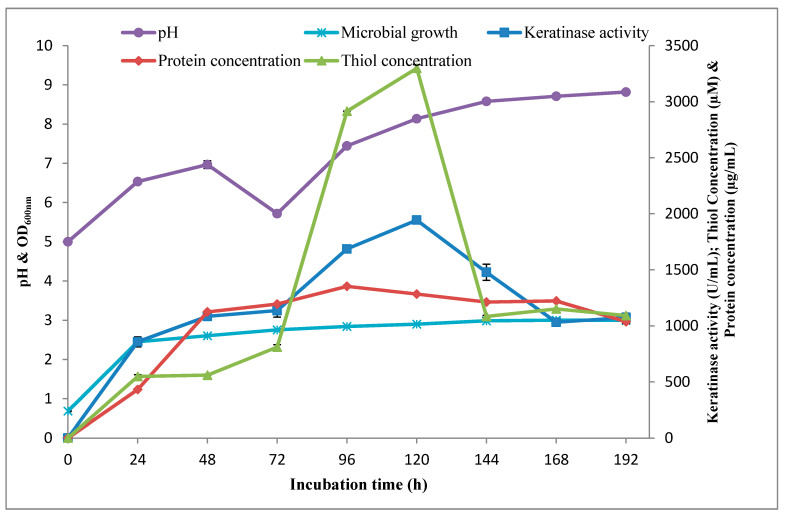
Time course profile of keratinolytic activity by *Bacillus* sp. Nnolim-K1 in an improved basal feather medium at 25 °C and 150 rpm. Each point represents the mean value of triplicate experiments.

The kinetics of keratinolytic activity by *Bacillus* sp. Nnolim-K1 was studied in an improved formulated medium. The extracellular keratinase production by *Bacillus* sp. Nnolim-K1 began at the exponential growth phase of strain Nnolim-K1 with a keratinase activity of 857.27 ± 44.99 U/mL at 24 h corresponding to the optical density of 2.45 ± 0.01, and increased consistently with increasing incubation time, and subsequently peaked at 120 h with a maximum keratinase activity of 1943.63 ± 0.0 U/mL (Figure 4). Beyond 120 h, enzyme activity decreased from 1474.18 ± 71.99 U/mL to 1073.64 ± 39.86 U/mL as the fermentation period increased from 144 h to 192 h, respectively. A similar result was reported for keratinase production by *B. megaterium* F7-1 [42] and *Xanthomonas* sp. P5 [45]. Decreased enzyme activity beyond optimum could be a result of the negative impact of accumulated metabolic byproducts and reduction in viable cell population [46]. 

The initial medium pH gradually increased from 5.0 to 6.97 ± 0.02 after 48 h of fermentation, and subsequently decreased to 5.72 ± 0.02 post 72 h cultivation. Thereafter, the pH increased consistently, reached a maximum value of 8.82 ± 0.0, after 192 h of fermentation. Cultivation medium of keratinolytic *B. licheniformis* ER-15 [16] and *Xanthomonas* sp. P5 [45], was reported to showcase similar pH fluctuation during the time course study. The drop in medium pH value could be due to accumulation of organic acids in the medium during the exponential growth phase; meanwhile, the shift of medium pH towards alkalinity has been attributed to the high concentration of ammonia, resulting from the deamination of amino-containing groups emanating from keratinolytic of chicken feather [16,44,47]. 

Furthermore, keratinolytic activity of *Bacillus* sp. Nnolim-K1 was accompanied by high medium thiol (sulfhydryl group) concentration (Figure 4). The thiol concentration gradually built up during the exponential growth phase, and climaxed at 120 h of fermentation, with a maximum concentration of 3298.15 ± 33.19 μM, after 120 h of cultivation. The concentration of thiols in the present study is significantly high when compared to the concentration of 2.89 mM formed in an unoptimized medium [20] and similar studies [5,6,45]. Notably, the pattern of thiol concentration coincided with that of keratinase production. Therefore, it will be prudent to state that *Bacillus* sp. Nnolim-K1 exhibited remarkable keratinolytic activity during cultivation in the feather-constituted medium [31]. The significant amount of thiol groups detected in the fermentation broth of *Bacillus* sp. Nnolim-K1 demands further investigation in order to unravel the origin of free sulfhydryl groups and the nature of the biodegradation products. 

### 3.2. Biochemical Characterization of Keratinase (KerBNK1) 

#### 3.2.1. Effect of pH and Temperature on KerBNK1 Activity and Stability

**Figure 5:**
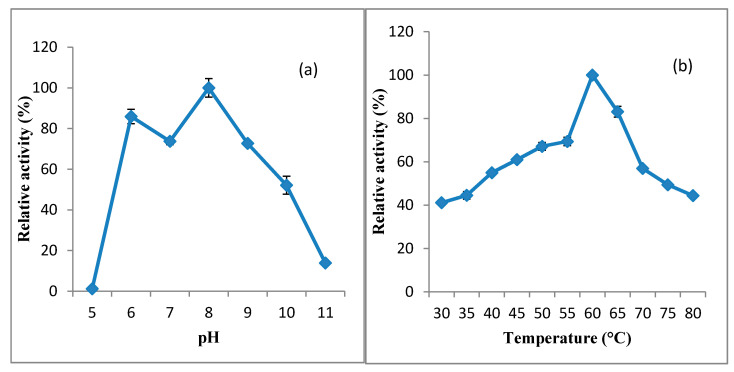
Effect of (**a**) pH and (**b**) temperature on KerBNK1 activity. Each point represents the mean value of triplicate assays.

The activity of KerBNK1 was determined in different buffer solutions ranging from pH 5.0 to pH 11.0. The results indicated that KerBNK1 was active from pH 6.0 to pH 10.0 with an optimal pH of 8.0 (Figure 5a). The enzyme had no activity at pH 5.0. KerBNK1 was active at pH 6.0 and slightly decreased at pH 7.0 with relative activity of 85.92 ± 3.52% and 73.74 ± 1.67% respectively. Beyond pH 8.0, the enzyme activity dropped consistently with relative activity of 72.69 ± 0.74%, 52.13 ± 4.45%, and 13.88 ± 0.37% at pH 9.0, 10.0 and 11.0, respectively. Bacterial keratinases are predominantly active in neutral to alkaline conditions, with an optimal pH ranging from 7.0 to 10.0 [12]. However, comprehensive reports on a few keratinases with an optimal activity at the weakly acidic condition or extremely alkalophilic condition abound [31,40]. The slight decline in enzyme activity at neutral pH may be as a result of altered protein side-chain protonation, and hence, slightly perturbed enzyme catalytic conformation.

Enzyme activity assay was carried out from 30 °C to 80 °C, and the results showed that KerBNK1 was optimally active at 60 °C (Figure 5b). At 30 °C, the enzyme had 41.14 ± 1.08% relative activity, and activity further increased with increasing temperature. Beyond optimal temperature (60 °C), the enzyme activity declined as temperature increased, with relative activities of 83.11 ± 2.49%, 56.93 ± 0.52%, 49.40 ± 0.80%, and 44.40 ± 0.99% at 65 °C, 70 °C, 75 °C, and 80 °C, respectively. A similar optimal temperature was reported for keratinase from *B. subtilis* RSE163 [48]. Likewise, two keratinases from *Bacillus halodurans* were optimally active at temperatures between 60 °C and 70 °C [49]. Keratinolytic proteases from variable bacterial strains have been reported to display temperature optima between 40 °C and 80 °C [12]. However, *Fervidobacterium islandicum* AW-1 produced thermophilic keratinase with an optimal temperature of 100 °C [33].

**Figure 6:**
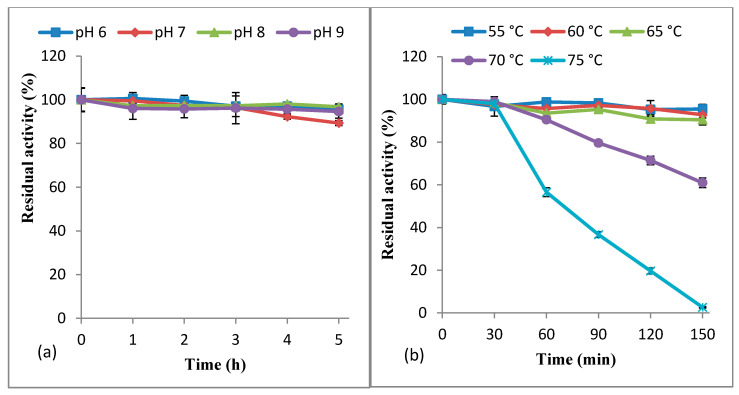
Effect of (**a**) pH and (**b**) temperature on KerBNK1 stability. Each point represents the mean value of triplicate assays.

From the stability study (pH 6.0–9.0), it was observed that KerBNK1 was highly stable in the various buffer solutions, retaining 95.61 ± 1.24%, 89.37 ± 1.14%, 96.76 ± 1.35%, and 94.62 ± 3.05% of the original activity at pH 6.0, 7.0, 8.0 and 9.0, respectively (Figure 6a). The keratinase from *B. subtilis* RSE163 demonstrated a similar pH stability pattern [48]. Conversely, KerBNK1 pH stability was superior to that reported for keratinase from *Bacillus altitudinis* RBDV1 [50].

We further investigated the thermal stability of KerBNK1 from 55 °C to 75 °C. The result showed that the enzyme was remarkably thermostable, maintaining the residual activity of 95.51 ± 1.27%, 92.82 ± 4.81%, 90.45 ± 0.80%, and 60.96 ± 2.27% after 150 min of preheating at 55, 60, 65, and 70 °C, respectively (Figure 6b). The enzyme retained 98.02 ± 0.54% and 56.57 ± 2.07% of the original activity after 30 min and 60 min of respective preheating, at 75 °C. Gupta et al. [48] reported alkaline keratinase with similar thermal stability. KerBNK1 showed higher thermal stability when compared to the previously characterized keratinases from *Bacillus* spp. [50,51]. The excellent pH and temperature stability demonstrated by KerBNK1 suggests its industrial and biotechnological application potentials. By the way, the crude enzyme was used for the study and the inherent impurities might have also contributed to the considerable stability demonstrated by KerBNK1 in the respective stability evaluations by conserving the catalytic integrity. Therefore, purification of the enzyme to relative homogeneity is imperative in order to reaffirm the present attributes of KerBNK1. 

#### 3.2.2. Effect of Chemical Agents and Metal Ions on KerBNK1 Stability

We evaluated the impact of chemical agents on the stability of KerBNK1. The results revealed that KerBNK1 was significantly stable in the presence of serine protease inhibitor (PMSF) with residual activity of 96 ± 2.62% (Table 3). Conversely, KerBNK1 completely lost activity in the presence of metal ions chelating agents; EDTA and 1,10-phenanthroline. This differential sensitivity to protease inhibitors indicated that KerBNK1 is a metallo-keratinase. Metal ion chelators remove metal cofactors that assist in the maintenance of enzyme catalytic orientation, hence promote loss of enzyme–substrate affinity and/or structural destabilization. The sensitivity of proteases to a different class of inhibitors is used to classify them, and comprehensive reports have described keratinases to predominantly belong to serine or metallo class of protease [12,30,40].

Furthermore, KerBNK1 exhibited variable stability in the presence of reducing agents tested, presenting remarkable residual activity of 102 ± 4.79% when pretreated with 2-mercaptoethanol (Table 3). On the contrary, sodium thioglycolate, dithiothreitol, and sodium sulfite negatively impacted KerBNK1 which recorded the respective residual activity of 76 ± 0.51%, 92 ± 0.29%, and 87 ± 0.65%. Acetonitrile, hydrogen peroxide, and propan-2-ol prompted a reduction of the original activity of KerBNK1, with respective residual activity of 91 ± 0.29%, 91 ± 2.69%, and 75 ±0.95%. Among the surfactants tested, triton X-100 and tween-80 promoted the catalytic efficiency of KerBNK1 with residual activity of 106 ± 2.54% and 119 ± 5.59%, respectively. Similarly, Riffel et al. [52] reported metallo-keratinase from *Chryseobacterium* sp. kr6 with significant stability in the presence of 2-mercaptoethanol and triton X-100. 

The stability of KerBNK1 in the presence of monovalent, divalent, and trivalent metal ions was evaluated. The results showed that KerBNK1 presented variable residual activity in different metal ions employed (Table 4). Ca^2+^ stimulated the catalytic efficiency of KerBNK1 with residual activity of 116 ± 5.16%. The marginal decline in enzyme activity, although not statistically significant when compared to the control, was observed with Li^+^, K^+^, Ag^+^, and Mg^2+^, presenting residual activity of 96 ± 1.96%, 94 ± 1.35%, 96 ± 1.59%, and 97 ± 3.56%, respectively. KerBNK1 stability was strongly affected in the presence of Na^+^, Zn^2+^, Cu^2+^, Ba^2+^, and Co^2+^, with residual activity of 76 ± 4.87%, 72 ± 0.95%, 77 ± 5.09%, 70 ± 3.19%, and 66 ± 5.67%, respectively. The overall observation shows that KerBNK1 could moderately tolerate chemical toxicity occasioned by metal ions, and may be catalytically stimulated when supplemented with an optimal concentration of Ca^2+^. *Bacillus pumilus* GRK crude keratinase had residual activity of 105.68 ± 0.40% in presence of 1 mM Ca^2+^ [18]. Likewise, metallo-keratinase from *Chryseobacterium* kr6 showed a similar pattern of stability as KerBNK1 with various metal ions tested [52]. On the contrary, *Microbacterium* sp. kr10 metallo-keratinase significantly lost activity in the presence of different metal ions employed; besides, complete activity inhibition was presented by 5 mM of Zn^2+^, Hg^2+^ and Cu^2+^ [53]. The observed stability of KerBNK1 when pretreated with various reducing agents, surfactants, and other chemical agents suggests the enzyme biotechnological and industrial prospects.

### 3.3. Molecular Characterization of KerBNK1

**Figure 7:**
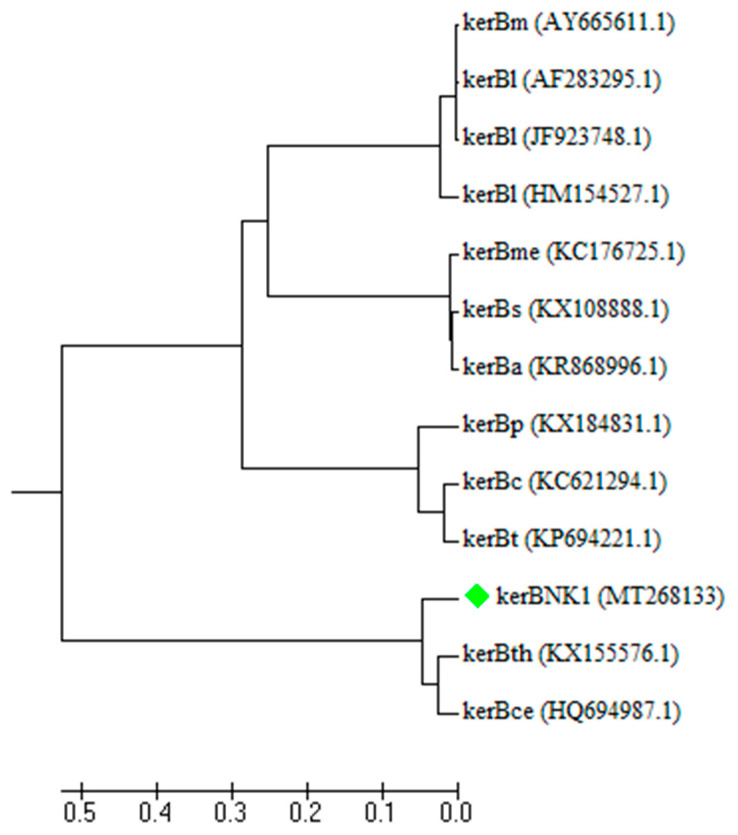
A phylogenetic tree of some selected keratinase genes of different *Bacillus* species was constructed in MEGA 7 and the evolutionary history was inferred using the unweighted pair group method with arithmetic mean (UPGMA). The tree is drawn to scale, with branch lengths in the same units as those of the evolutionary distances used to infer the phylogenetic tree. The keratinase genes (*ker*) considered in this study were from; Bm: *Bacillus mojavensis*; Bl: *Bacillus licheniformis*; Bme: *Bacillus methylotrophicus*; Bs: *Bacillus subtilis*; Ba: *Bacillus amyloliquefaciens*; Bp: *Bacillus Pumilus*; Bc: *Bacillus circulans*; Bt: *Bacillus tequilensis;* Bth: *Bacillus thuringiensis;* Bce: *Bacillus cereus*. The keratinase gene (*kerBNK1*) from *Bacillus* sp. Nnolim-K1 is indicated with a squared green tip. The GenBank accession numbers are shown in parenthesis.

The putative keratinase-encoding gene (*kerBNK1*) amplification yielded a PCR product with a band size of 1104 bp (Supplementary Figure S1). The GenBank was searched for similar sequences as *kerBNK1* and the result showed that *kerBNK1* had 95.38% and 95.21% of sequence similarity with keratinase genes from *Bacillus cereus* Wu6 (*ker6*; accession number: HQ694987) and *Bacillus thuringiensis* S3KUBOT (*ker-S3KUBOT*; accession number: KX155576), respectively. Additionally, it showed 95.1% of sequence similarity with *Bacillus cereus* ATCC 4342 Subtilisin Carlsberg gene (KFM89048). The partial nucleotide sequence of *kerBNK1* showed a G+C content of 36.3%, and was submitted to the GenBank with the accession number MT268133. A few keratinases of bacteria origin have been sequenced so far, of which the majority was from *Bacillus* species [11]. To date only about two keratinase genes of *B. cereus* sensu lato group have been successfully sequenced and deposited in GenBank. This group of *Bacillus* species has been reported to display excellent keratinolytic activity toward degradation of keratinous biomass [19]. The phylogeny of *kerBNK1* and other keratinase gene sequences of different groups of *Bacillus* spp. are presented in Figure 7. The dendrogram showed the distribution of the keratinase genes into four distinct clades, hence, inferring their evolutionary history. Keratinase genes of *B. licheniformis* (*kerBl*) clustered with *Bacillus mojavensis* (*kerBm*). Similarly, keratinase genes from *Bacillus methylotrophicus* (*kerBme*), *B. subtilis* (*kerBs*), and *B. amyloliquefaciens* were under a common clade. The third clade was made up of keratinase genes from *B. pumilus* (*kerBp*), *Bacillus circulans* (*kerBc*), and *Bacillus tequilensis* (*kerBt*). The last clade consisted of *kerBNK1* and keratinase genes from *B. thuringiensis* (*kerBth*) and *B. cereus* (*kerBce*). Notably, the *B. cereus* group cluster is uniquely rooted in the phylogenetic tree as an out-group. This may indicate the level of variations among these keratinase-encoding genes. Therefore, the evolutionary relationship between the different clusters of keratinase genes, especially with the *B. cereus* group of which *kerBNK1* is a member, suggests an evolutionary divergence. 

Translation of partial sequence of *kerBNK1* gave a keratinase sequence (KerBNK1) with 171 amino acid residues. Further analysis of the deduced partial KerBNK1 sequence showed a molecular weight, isoelectric point, instability index, and aliphatic index of 19.37 kDa, 6.03, 22.69, and 60.35, respectively. Molecular weight determined from the sequence of KerBNK1 was low in relation to other characterized keratinases of Bacillus origin [40]. This was expected as the partial sequence of KerBNK1 was analyzed. Nonetheless, keratinases with a molecular weight of 17 kDa and 18 kDa have been previously characterized [54,55]. Likewise, the partial sequence could have prompted the relatively low value of the theoretical isoelectric point of KerBNK1, considering that the enzyme was optimally active at alkaline pH. Notably, binding of metal ions on a protein could significantly increase the pI value of a protein [56]. Hence, this could be the case for the metallo-keratinase under investigation, since the pI of proteins theoretically suggests their pH optima [57].

**Figure 8:**
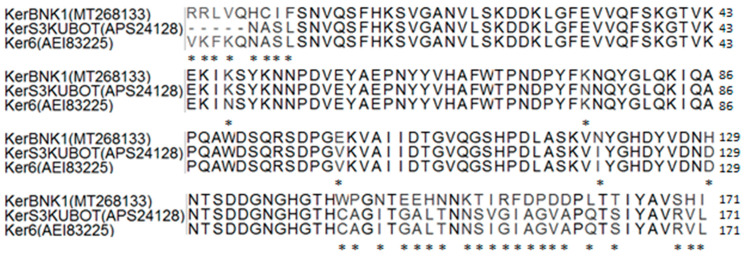
Amino acid sequence alignment of KerBNK1 with the keratinases of *Bacillus thuringiensis* S3KUBOT (KerS3KUBOT) and *Bacillus cereus* Wu6 (Ker6). *indicates variations in the aligned enzyme sequence residues.

The instability index computed for KerBNK1 showed that the enzyme is structurally stable in nature (instability index <40) [28,57]. The aliphatic index indicates the degree of thermal stability of a protein and this is wholly dependent on the relative volume of a protein occupied by the aliphatic residues [58]. The aliphatic index determined for KerBNK1 implied that the enzyme is significantly thermostable [57]. Multiple sequence alignment of KerBNK1 with other keratinase sequences from the *B. cereus* group (sensu lato) showed some variations of amino acid residues and the points of dissimilarity among the sequences are indicated with asterisks (*; Figure 8). The sequence homology of KerBNK1 with other related enzymes confirmed that *Bacillus* sp. Nnolim-K1 produced keratinase that mediated the chicken feather degradation. However, the disparities among the aligned sequence residues suggest that KerBNK1 is a novel keratinase, and consequently, may be amply endowed with unique potentials relevant to many sectors of bioeconomy. Importantly, the unique nature of KerBNK1 may have contributed to the short timeline of chicken feather bioconversion by *Bacillus* sp. Nnolim-K1. Therefore, the attributes of *Bacillus* sp. Nnolim-K1 and the keratinase underpin their immense potentials towards sustainable green technology. 

### 3.4. Impact of Laundry Detergent on KerBNK1 Stability

**Figure 9:**
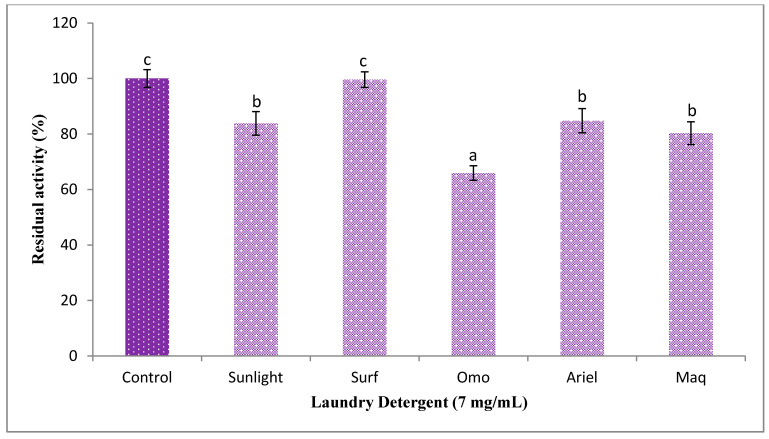
Impact of laundry detergents on KerBNK1 stability. a, b and c indicate statistically significant difference among the different tests; and treatments without similar letter(s) are significantly different (*p* < 0.05).

The results of KerBNK1 stability evaluation showed that the enzyme was significantly stable, although in a variable fashion, in different detergents employed, achieving residual activity of 65.91 ± 2.64%, 80.28 ± 4.14%, 83.81 ± 4.28, 84.77 ± 4.35% and 99.59 ± 2.78% in the presence of Omo, Maq, Sunlight, Ariel and Surf respectively (Figure 9). Similarly, crude keratinase from *B. pumilus* GRK retained 89.48 ± 0.22% of the original activity after 60 min of Ariel pretreatment [18]. Under similar pretreatment conditions, KerBNK1 showed higher stability in the presence of Ariel against SAPB from *B. pumilus* CBS that retained about 67% of the original activity [59]. Furthermore, the stability of KerBNK1 was superior to that reported for crude keratinase from *Paenibacillus woosongensis* TKB2, where 48.1–70.4% of the original activity was retained 60 min post detergent pretreatment [17]. The degree of impact of the various laundry detergents on the enzyme under investigation might be ascribed to variations in the respective detergent ingredients, and such variations have been reported to affect the stability of detergent endogenous enzymes [18]. The remarkable stability exhibited by KerBNK1 suggests its promising candidacy as an excellent bio-additive in detergent formulation. 

### 3.5. Biodegradation of White and Melanized Feathers–Structural Study 

**Figure 10:**
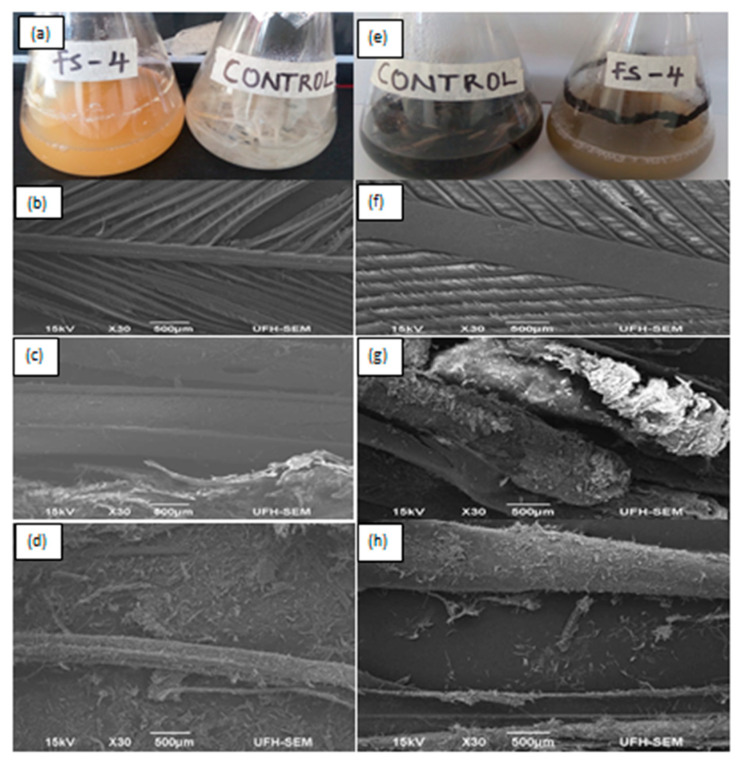
Feather degradation by *Bacillus* sp. Nnolim-K1: cultivation and control flasks of (**a**) white feather and (**e**) melanized feather. Electron micrographs of uninoculated (**b**) white feather, (**f**) melanized feather, (**c**,**g**) 24 h postinoculation, and (**d**,**h**) 48 h postinoculation.

Figure 10a,e show biodegradation of white and melanized feathers by *Bacillus* sp. Nnolim-K1 after 48 h of fermentation. Based on the weight loss approach, the degree of feather degradation after 48 h was determined to be 93.87 ± 1.41% and 91.95 ± 1.34% for the white and melanized feathers, respectively. The structural study of the degraded feathers via SEM showed complete detachment of barbules of white and melanized feathers after 24 h (Figure 10c,g) when compared to the controls (Figure 10b,f). Remarkably, structural distortion of white and melanized feathers rachis was observed after 48 h of fermentation (Figure 10d,h). Similarly, *Chryseobacterium* sp. RBT achieved 83% degradation of melanized feather after 48 h of incubation [8]. *Bacillus altitudinis* GVC11 significantly dismembered white and melanized feathers after 48 h and 96 h of cultivation, respectively [37]. The prolonged degradation of the melanized feather was attributed to pigmentation. Contrariwise, keratinolytic *B. megaterium* F7-1 [42] and *B. licheniformis* PWD-1 [6] displayed remarkable feather degradation after 7 and 10 days of fermentation, respectively. Remarkably, efficient feather degradation has been reported for commercially available protease–Ronozyme ProAct from *Norcardiopsis prasin* after 16 h of incubation [60]; although the degradation process was facilitated with a reducing agent–sodium thioglycolate (2%). Therefore, the ability of *Bacillus* sp. Nnolim-K1 to significantly degrade white and melanized feathers within a short time period underpins its application potentials in the bioconversion of keratinous agro-wastes into useful products. Future evaluation of the keratin degradation potential of the keratinase from *Bacillus* sp. Nnolim-K1 is imperative, in order to understand whether the keratin hydrolysis is executed by the keratinolytic protease alone, or in synergy with other microbial cell-associated reducing agents such as sulfites and reductases.

## 4. Conclusion

In conclusion, *Bacillus* sp. Nnolim-K1 demonstrated remarkable keratinolytic activity in a relatively cheap medium, at mesophilic conditions, and moderate agitation speed. The stability of the keratinase at broad pH and temperature, chemical agents, and laundry detergents suggests its biotechnological and industrial application potentials. The ability of *Bacillus* sp. Nnolim-K1 to degrade white and melanized feathers within a short time interval underpins its potential biotechnological relevance in valorizing agro-wastes into valuable products. Therefore, further study on the profiling of feather fermentation broth is imperative, so as to determine the nature of biodegradation products. The analysis of the amplified keratinase gene fragment that codes KerBNK1 showed a novel amino acid sequence. Hence, cloning and overexpression of this gene into an industrially suitable host is exigent, and would be implemented for scale-up production of this important biocatalyst.

## Figures and Tables

**Table 1:** Experimental ranges of four parameters used in the central composite design of response surface methodology (RSM).

Code Value	Independent Variables	Range Coding Level		
		−1	0	+1
A(X_1_)	Xylose (%; *w/v*)	0.4	0.6	0.8
B(X_2_)	pH	5.0	6.0	7.0
C(X_3_)	Feather (%; *w/v*)	1.0	1.25	1.5
D(X_4_)	Inoculum (%; *v/v*)	1.0	2.0	3.0

**Table 2:** Central composite design (CCD) design of RSM using four independent variables showing actual and predicted values of keratinase activity by *Bacillus* sp. Nnolim-K1.

Run	Independent Variable				Response: Keratinase Activity (U/mL)	
	A: Xylose (%)	B: pH	C: Feather (%)	D: Inoculum (%)	Experimental	Predicted
1	0.6(0)	7(+1)	1.25(0)	2(0)	1181.82	1180.66
2	0.6(0)	6(0)	1.25(0)	2(0)	1280.91	1232.85
3	0.8(+1)	5(−1)	1(−1)	1(−1)	1320.91	1313.14
4	0.6(0)	6(0)	1.25(0)	1(−1)	1217.27	1209.21
5	0.8(+1)	5(−1)	1(−1)	3(+1)	1589.09	1460.64
6	0.6(0)	5(−1)	1.25(0)	2(0)	1367.27	1344.30
7	0.4(−1)	6(0)	1.25(0)	2(0)	1219.09	1162.38
8	0.8(+1)	5(−1)	1.5(+1)	3(+1)	1332.73	1249.40
9	0.8(+1)	6(0)	1.25(0)	2(0)	1146.36	1178.94
10	0.8(+1)	7(+1)	1(−1)	3(+1)	1217.27	1297.00
11	0.8(+1)	7(+1)	1.5(+1)	3(+1)	1145.45	1085.76
12	0.6(0)	6(0)	1.25(0)	2(0)	1369.09	1232.85
13	0.4(−1)	5(−1)	1(−1)	1(−1)	1253.64	1296.57
14	0.6(0)	6(0)	1.25(0)	2(0)	1318.18	1232.85
15	0.8(+1)	7(+1)	1(−1)	1(−1)	1130.91	1149.50
16	0.6(0)	6(0)	1.25(0)	2(0)	1164.54	1232.85
17	0.4(−1)	7(+1)	1(−1)	1(−1)	1146.36	1132.93
18	0.4(−1)	5(−1)	1.5(+1)	3(+1)	1213.64	1232.83
19	0.8(+1)	7(+1)	1.5(+1)	1(−1)	1145.45	1138.72
20	0.4(−1)	7(+1)	1(−1)	3(+1)	1369.09	1280.43
21	0.6(0)	6(0)	1.5(+1)	2(0)	1253.64	1218.34
22	0.6(0)	6(0)	1(−1)	2(0)	1318.18	1329.35
23	0.6(0)	6(0)	1.25(0)	2(0)	1312.73	1232.85
24	0.4(−1)	5(−1)	1.5(+1)	1(−1)	1346.36	1285.79
25	0.4(−1)	5(−1)	1(−1)	3(+1)	1358.18	1444.07
26	0.6(0)	6(0)	1.25(0)	2(0)	1070.91	1232.85
27	0.4(−1)	7(+1)	1.5(+1)	1(−1)	1146.36	1122.15
28	0.8(+1)	5(−1)	1.5(+1)	1(−1)	1147.27	1302.35
29	0.6(0)	6(0)	1.25(0)	3(+1)	1080.91	1256.49
30	0.4(−1)	7(+1)	1.5(+1)	3(+1)	973.64	1069.20

**Table 3:** Effect of chemical agents on KerBNK1 stability.

Chemical Agent	Concentration	Residual Activity (%)
None	-	100 ± 3.92
EDTA	5 mM	0 ± 0
1,10-phenanthroline	5 mM	0 ± 0
PMSF	5 mM	96 ± 2.62
Sodium thioglycolate	5 mM	76 ± 0.51
Dithiothreitol	5 mM	92 ± 0.29
Sodium sulfite	5 mM	87 ± 0.65
2-Mercaptoethanol	5 mM	102 ± 4.79
Hydrogen peroxide	1% (*v/v*)	91 ± 2.69
Acetonitrile	1% (*v/v*)	91 ± 0.29
Propan-2-ol	1% (*v/v*)	75 ±0.95
Sodium dodecyl sulfate	5 mM	84 ± 1.53
Triton X-100	1% (*v/v*)	106 ± 2.54
Tween-80	1% (*v/v*)	119 ± 5.59

The values are presented as the mean and standard deviation of triplicate assays.

**Table 4:** Effect of metal ions on KerBNK1 stability.

Metal Ion	Concentration (mM)	Residual Activity (%)
None	-	100 ± 3.92
Li^+^	5	96 ± 1.96
Na^+^	5	76 ± 4.87
K^+^	5	94 ± 1.35
Ag^+^	5	96 ± 1.59
Ca^2+^	5	116 ± 5.16
Mg^2+^	5	97 ± 3.56
Mn^2+^	5	91 ± 1.38
Zn^2+^	5	72 ± 0.95
Cu^2+^	5	77 ± 5.09
Fe^2+^	5	86 ± 2.18
Hg^2+^	5	82 ± 0.07
Ba^2+^	5	70 ± 3.19
Co^2+^	5	66 ± 5.67
Al^3+^	5	89 ±2.83
Fe^3+^	5	77 ± 0.65

The values are presented as the mean and standard deviation of triplicate assays.

## Supplementary Materials

The following are available online at https://www.mdpi.com/2076-2607/8/9/1304/s1, Figure S1: Gel picture of the keratinase gene amplification from *Bacillus* sp. Nnolim-K1.
