# Supplementary material for: Biochemical and Molecular Characterization of a Thermostable Alkaline Metallo-Keratinase from Bacillus sp. Nnolim-K1
Source: Microorganisms. 2020 Aug 27;8(9):1304. doi: 10.3390/microorganisms8091304 (PMC7565512; doi:10.3390/microorganisms8091304)
Supplement: Supplementary file 1 [file microorganisms-08-01304-s001.pdf]

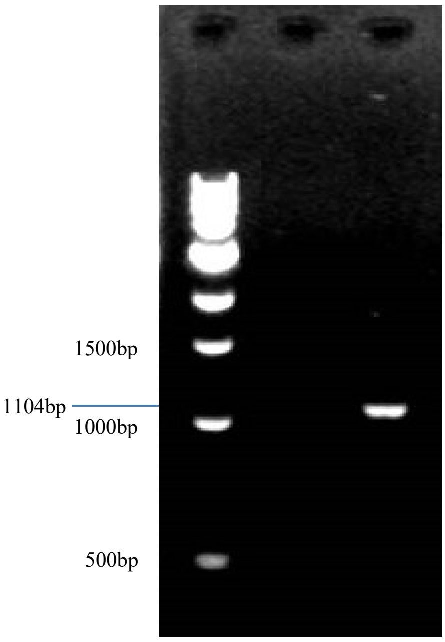

Figure S1. Gel picture of the keratinase gene amplification from *Bacillus* sp. Nnolim-K1. Lane 1: DNA marker; lane 2: negative control; lane 3: keratinase gene (1104 bp).
